# Group or ungroup – moose behavioural response to recolonization of wolves

**DOI:** 10.1186/s12983-017-0195-z

**Published:** 2017-02-17

**Authors:** Johan Månsson, Marie-Caroline Prima, Kerry L. Nicholson, Camilla Wikenros, Håkan Sand

**Affiliations:** 1 Department of Ecology, Grimsö Wildlife Research Station, Swedish University of Agricultural Sciences, SE-730 91 Riddarhyttan, Sweden; 2 Département de Biologie, 1045, av de la Médecine, Université Laval, Québec, G1V 0A6 Canada

**Keywords:** *Alces alces*, anti-predator, Behaviour, *Canis lupus*, Group size, Predator, Prey, Ungulate

## Abstract

**Background:**

Predation risk is a primary motivator for prey to congregate in larger groups. A large group can be beneficial to detect predators, share predation risk among individuals and cause confusion for an attacking predator. However, forming large groups also has disadvantages like higher detection and attack rates of predators or interspecific competition. With the current recolonization of wolves (*Canis lupus*) in Scandinavia, we studied whether moose (*Alces alces*) respond by changing grouping behaviour as an anti-predatory strategy and that this change should be related to the duration of wolf presence within the local moose population. In particular, as females with calves are most vulnerable to predation risk, they should be more likely to alter behaviour.

**Methods:**

To study grouping behaviour, we used aerial observations of moose (*n* = 1335, where each observation included one or several moose) inside and outside wolf territories.

**Results:**

Moose mostly stayed solitary or in small groups (82% of the observations consisted of less than three adult moose), and this behavior was independent of wolf presence. The results did not provide unequivocal support for our main hypothesis of an overall change in grouping behaviour in the moose population in response to wolf presence. Other variables such as moose density, snow depth and adult sex ratio of the group were overall more influential on grouping behaviour. However, the results showed a sex specific difference in social grouping in relation to wolf presence where males tended to form larger groups inside as compared to outside wolf territories. For male moose, population- and environmentally related variables were also important for the pattern of grouping.

**Conclusions:**

The results did not give support for that wolf recolonization has resulted in an overall change in moose grouping behaviour. If indeed wolf-induced effects do exist, they may be difficult to discern because the effects from moose population and environmental factors may be stronger than any change in anti-predator behaviour. Our results thereby suggest that caution should be taken as to generalize about the effects of returning predators on the grouping behaviour of their prey.

**Electronic supplementary material:**

The online version of this article (doi:10.1186/s12983-017-0195-z) contains supplementary material, which is available to authorized users.

## Background

Predation risk influences the adoption of potentially costly anti-predatory behaviour by prey. To avoid predation, prey may modify their vigilance, habitat selection, movement patterns, spatial and temporal distribution, or sexual segregation [1–4]. However, prey cannot be solely devoted to predator avoidance behaviours as they are obligated to obtain necessary resources for growth, reproduction, and survival. Therefore anti-predator adaptations need to be balanced against the present risk level [1]. For instance, a spatio-temporal variation in predation risk may lead to prey adopting different behavioural strategies including more pronounced anti-predator behaviour in certain areas or during periods of higher relative risk [5].

Predation risk is thought to be one of the primary motivations for animals to change their grouping strategy e.g., form larger groups or avoid conspecifics and thereby decrease group size [6–10]. The choice of strategy is context- and species dependent and a general pattern seems to be that prey in open terrain aggregate more while prey in closed environments are more solitary [9]. As group size increases, there are more eyes scanning the environment, more individuals will share the predation risk among several group members and more individuals may also cause confusion to the predator when being attacked [1, 11, 12]. With increased group size any one individual forager can devote less time to vigilance and more time to feeding [13–15]. However, there are also negative consequences of forming groups as larger groups may imply increased risk of detection and attack rate by predators [16, 17]. Forming larger groups may also increase competition over critical resources [18–20]. As a consequence, an alternative strategy to minimize predation risk can also be to form small groups that rarely are encountered by predators [17]. Moreover, the net gain of forming groups is dependent on the individual’s condition and placement in the group; for example low status and marginal individuals in the group may have a higher predation risk [10, 21]. Individuals may therefore respond differently to variation in predation risk according to the costs and benefits associated with grouping behaviour. It is therefore expected that prey individuals should show different grouping behaviour in response to the presence of predators but also that this may be linked to the individual’s vulnerability to predation.

Prey vulnerability to predation can be directly linked to the characteristics of the predator and predator population (such as search image, and population density) but also to individual traits and physical condition of the prey and to environmental conditions [22–24]. Several studies have reported higher risk for low ranked (e.g., immature and small-sized) individuals [24–26] and increased risk during times of harsh climatic conditions as for example deep snow cover [22, 25, 27, 28]. Heard [23] showed that musk-ox (*Ovibos moschatus*) group size increased with an increase of wolf (*Canis lupus*) density but also that group size was dependent on the prey type targeted by wolves (i.e., larger groups during seasons when wolves preyed more on musk-ox). Moreover, an individual’s status or potential ranking within a group can necessitate formation or avoidance of grouping behaviour [18]. For instance, groups formed by individuals with the same physical condition may benefit from dilution and distribute the probability of capture amongst group members. In contrast, a low ranked individual may benefit from the many eyes and overall increased vigilance in larger groups. Conversely, a low ranked individual could be at disadvantage as it is considered a marginal individual in the group and may be exposed to competitive disadvantage for resources [29, 30]. It is therefore not always easy to predict which individual that will obtain most benefits by group formation.

Among ungulates, the tendency to form groups varies between species, with variation in population density, food distribution, and between habitats [31, 32]. Moose (*Alces alces)* have been described as a “quasi-solitary” species since they show both solitary and group living behaviour, possibly as a response to increased predation risk in open habitats [2, 18, 33]. For instance, in Alaska, moose were found to form larger groups at greater distance from cover, which suggests that social grouping in moose, in addition to other factors e.g., rutting and mobility in deep snow, is an adaptation to increased predation risk [18].

Starting in the early 1980s moose have been re-exposed to predation risk from wolves, in south-central Scandinavia, as wolves re-colonized this region after being extinct for more than 100 years [34]. Here, we study whether moose grouping behaviour change as a response to the recolonization of wolves. Moose in Scandinavia are related to forested areas i.e., closed environments. According to earlier studies on browsing deer (concentrate selectors) and ungulates living in terrain with cover, these species are less prone to form larger groups due to both foraging and/or antipredator behaviour [9, 10, 35]. It’s therefore not obvious if and how moose should change their grouping behaviour when predation risk increase. However, if the solitary pattern of moose has been relaxed due to the period without wolves a decrease rather than an increase in group size may be expected. Moose can deploy both flight and fight strategies to avoid predation [36] but do not form defensive formations as groups of musk-oxen and mule deer [10, 37] which also support the prediction that moose will not form larger groups when re-exposed to predation. Here, we test whether moose grouping behaviour changes as a response to the recolonization of wolves. If a change in grouping behaviour exist, we predict that females with calves would be most prone to change their behaviour because calves of the year are the main prey by wolves in Scandinavia [38, 39]. In addition, we predict that if the presence of wolves is important for the grouping behaviour of moose, the strength of this behaviour would be linked to the duration of wolf presence (i.e., time since territory establishment). Moose within recently established territories may therefore express a less pronounced change in grouping behaviour than moose that has been exposed to wolves for a longer time period. In addition to the potential effects of wolf predation risk, we also considered population and environmental variables that may influence grouping behaviour such as moose density, adult sex-ratio and snow depth.

## Methods

### Study area

**Fig. 1:**
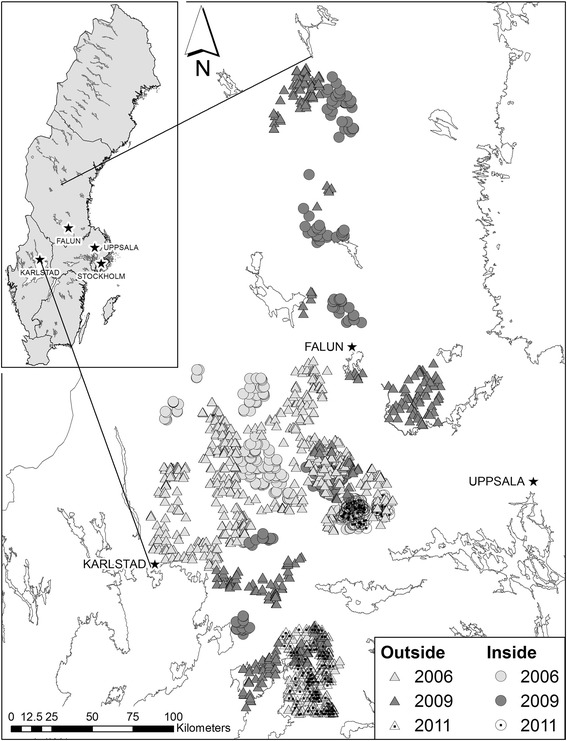
The study area in south-central Sweden with the distribution of aerial moose observations and whether they were classified as inside or outside wolf territories. Note, that some areas can be considered as both inside and outside due to the successive establishment of new wolf territories

The study area encompasses approximately 50 000 km^2^ within the boreal zone of south-central Sweden (Fig. 1, 58.58°-62.16 °N, 13.45°-16.64 °E). The area mainly consists of forests mixed with agriculture fields, bogs and lakes. Forests are dominated by Norway spruce (*Picea abies*) and Scots pine (*Pinus sylvestris*) mixed with deciduous trees, such as birch (*Betula spp.*), aspen (*Populus tremula*), alder (*Alnus incana*) and willow (*Salix spp*), and is intensively managed for timber and pulp. Mature stands are harvested by clear-cutting and reforested by planting or natural regeneration, resulting in an even-aged forest stand mosaic. Average monthly temperature range between +15 °C and -5 °C with the coldest month in January and the warmest month in July [40]. The ground is usually snow covered between late November and early April with a mean snow depth of 20 cm in mid-January [41].

### Studied populations

Moose populations are managed in Sweden and approximately 25-30% of the population is harvested annually [42]. Management in many areas is female biased to promote high productivity [42]. Moose density during winter commonly ranged between 0.6 and 2.5 moose/km^2^ [43, 44]. Roe deer (*Capreolus capreolus*) are distributed over the whole study area at variable densities, whereas red deer (*Cervus elaphus*) and wild boar (*Sus scrofa*) only occur in scattered populations in the south [45].

In 1983, wolves started a re-colonization of Scandinavia and has increased in numbers and distribution since the early 1990s [34, 46]. The wolf distribution area in Scandinavia currently covers approximately 100 000 km^2^ [47]. In the winter of 2010/2011 the population consisted of 31 family groups and 30 scent-marking pairs [48]. Brown bear (*Ursus arctos*) also occur at variable densities within our study area and prey mainly on natal moose calves during early summer [49].

### Moose aerial counts

We used aerial surveys made by helicopter to obtain data on the number and spatial distribution of moose in the landscape. These data were further used for estimating the size of moose groups, the relative density and adult sex ratio of moose. Aerial surveys were conducted in 2006, 2009 and 2011 by Svensk Naturförvaltning (www.naturforvaltning.se) in order to estimate moose densities that could be used for estimating appropriate hunting quotas. The moose is counted by transect survey and two different methods were used to subsample surveyed area; “square sampling” (782 observations) and “distance sampling” (553 observations), including a total of 1335 observations where each observation included one or several moose. For both survey methods, line transects were used and the sampling method used unlikely influence the observed grouping behaviour by moose. Moose surveys were conducted from mid-December to mid-February during short time periods with snow cover in order to increase detectability (for detailed description of aerial counts and distance sampling; [50, 51]. For each moose observation, the location was recorded by GPS (±10 m accuracy) and all moose classified according to age class (calves or adults) and sex.

### Presence of wolves and territory range

Presence/absence data of wolves for each area of aerial sampling was obtained by the ongoing annual monitoring of the Scandinavian wolf population throughout the study period (Liberg et al. 2012). Snow tracking in combination with DNA-analysis of scats and oestrus bleedings are used to monitor wolf family groups and scent-marking pairs which also gives a minimum size of territorial wolves [52]. In addition to snow tracking, several wolves were radio-collared (VHF and GPS) during the study period [53]. Seventeen wolf territories were included in the study and were defined by applying a 100% minimum convex polygon (MCP; [54]) on either locations from collared wolves or information collected from repeated snow tracking events in an area. Only scent-marking pairs and family groups were included to ensure that moose had regularly experienced wolf presence. Because the establishment of all wolf territories in Scandinavia has been annually registered since the founding of the population in 1983, the duration, i.e., the number of years, that moose had been exposed to territorial wolves could be estimated for each wolf territory and ranged from 1 to 13 years. Because non-collared and some of the collared wolves were monitored during a limited portion of the year, we buffered the estimated boundary of these wolf territories using a conservative approach to ensure that moose observations actually were outside any wolf territory (see below for classification of moose observations). We generated buffer zones by using two different methods because accuracy differs between MCPs estimated by snow tracking versus radio-tracking data. For MCPs (*n* = 10), based on snow-tracking data, we added a buffer zone so that the buffered territory equalled the maximum potential wolf territory observed in Scandinavia [1700 km2 based on GPS-data; ,53]. For MCPs based on radio-tracking data (*n*_total_ = 7; 2VHF and 5GPS), for which we have less than 12 months of data (*n* = 3; data range 1-7 months), we generated buffer zones so that the total area corresponded to one year of radio-tracking (see [53] for the proportion of annual home range covered in relation to studied time period).

### Classification of moose observations and estimation of snow depth

Moose observations were classified as inside wolf territories (presence of wolves), outside wolf territories (absence of wolves), or in a buffer zone (uncertain wolf presence). Observations inside the buffer zone were excluded from the analysis as were any moose observation with missing data on sex and age. All adult moose observed within 100 m from each other were considered as a group, i.e., one group can consist of one or several moose [55, 56]. Calves were not included in the group size estimate because of their propensity to move with their mother (not independent). Moose observations were classified in 5 categories depending on the composition of the group: 1) females + calves; 2) mixed group + calves; 3) mixed group no calves; 4) females; 5) males. Adult sex-ratio of moose was estimated both inside and outside wolf territories as the proportion of females in the adult (>1 year) population. The average snow depth was measured to the nearest centimetre (range 0-75) at randomly selected sites where the helicopter could land during the aerial count (i.e., open areas in the terrain) using a metre stick.

### Statistical analysis

We used winter aerial survey data of moose spatial distribution and group size to investigate if moose within wolf territories employed different grouping strategies as compared to moose in areas not yet re-colonized by wolves. We used a generalized linear model with a Poisson distribution to model group size as a function of the variables of interest [57]. We tested the spatial autocorrelation of the response variable with a permutation Moran’s test [58]. Two observations (group size) were considered as spatially auto-correlated if they were within 16 km which should be considered as conservative as it equals a distance ten times the radius of winter home range of moose [59]. The test showed a significant spatial autocorrelation in adult moose group size (Moran’s *I* = 0.04, *p*-value = 0.001). Therefore to take into account and correct for spatial autocorrelation, we computed the local Moran’s index [60] for each observation using the same neighbourhood weights matrix and included this as an explanatory variable in all the models. We defined a set of models including our five explanatory variables (i.e., either wolf absence/presence or time since wolf territory establishment, moose density, snow depth, adult sex ratio, spatial autocorrelation) and identified the most parsimonious model based on Akaike Information Criteria (AIC) and AIC weight (ωi; [61]). Then model fit was assessed using the pseudo R^2^ [57].

We conducted two separate analyses using the same model set. First, the response variable was the group size of adult moose (all categories together). Second, we considered each moose group category (listed above) separately and evaluated the explanatory variables. When considering each category separately, moose group size was no longer spatially auto-correlated, therefore we did not include this variable in this part of the analysis. Finally, we used multi-model inference from the top-ranked models (ΔAIC <2) to estimate coefficients and 85% confidence intervals. Because over dispersion parameters (ĉ) ranged 0.17 – 0.88 we did not have to correct for this [57]. Adding the variable year of survey as a random effect did not increase model fit (i.e., variation in AIC between the model with random effect and the model without random effect <2) and was therefore excluded from further analysis.

## Results

Of the total 1335 moose observations, 383 were inside wolf territories and 952 were outside (Table 1). Moose density inside and outside wolf territories averaged 1.08 (±3.43 S.D.) moose/km^2^ and 1.00 (±3.89) moose/km^2^, respectively. The proportion of females among adult moose ranged 0.58 – 0.64 inside and 0.62 – 0.69 outside wolf territories (over the three years included in the study).

**Table 1:** Total number of moose groups observed, total number of moose and group size range for each group category of moose in Sweden from aerial counts 2006, 2009, and 2011 inside (In) and outside (Out) wolf territories

Category	Females + calves		Mixed group no calves		Mixed group + calves		Females		Males	
	In	Out	In	Out	In	Out	In	Out	In	Out
Groups (n)	148	425	64	111	27	87	75	173	69	156
Adult moose (n)	170	471	228	378	80	250	109	239	145	235
Group size range	1-5	1-5	2-9	2-8	2-7	2-9	1-4	1-4	1-8	1-5

**Fig. 2:**
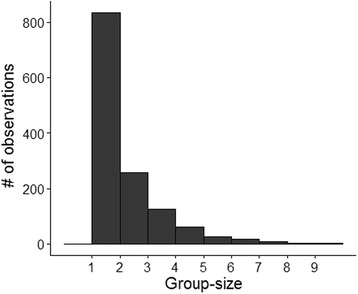
Distribution of adult moose group size in Sweden from aerial survey data collected in 2006, 2009, and 2011

**Fig. 3:**
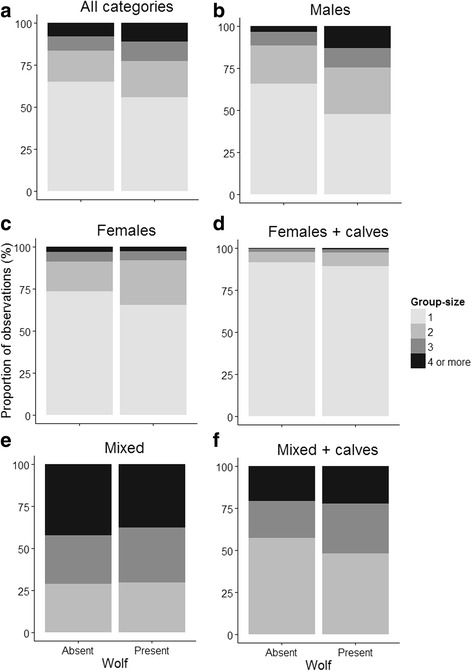
Proportion of group size observations of moose inside (wolf present) and outside (wolf absent) wolf territories by moose group category (**a**-all categories, **b**-males, **c**-females, **d**-females+calves, **e**-mixed, **f**-mixed with calves) from aerial survey data collected in Sweden in 2006, 2009, and 2011

The median group size inside and outside wolf territories were both equal to 1. Overall, smaller groups were more common than larger groups and 82% of the observations consisted of groups with less than three adult moose (Fig. 2). Moreover, groups composed of a mix of females and males were the most gregarious category with 70% of the groups including three or more adult moose (Fig. 3). Females with calves were the most common group category both inside and outside wolf territories (Table 1) but also the least gregarious category (Fig. 3). This category had a minimum group size of 1 adult and a maximum group size of 5 adults (Table 1) but more than 90% of the observations contained groups of only 1 adult female (Fig. 3c) and was not dependent on wolf presence. In both unisex and mixed groups, the presence of calves was linked to smaller groups (Fig. 3).

We evaluated 12 *a priori* models in order to explain moose grouping behaviour (Additional file 1: Table S1). The best model from our candidate models list included moose density, snow depth, and adult sex-ratio (Table 2). This model explained 13% of the variation in adult moose group size (pseudo R^2^ = 0.13). Wolf presence was not included in the best model. Including a variable for the time since wolf territory establishment did not increase model fit (i.e., difference in AIC between the model with wolf presence and the model with number of years since wolf territory establishment <2). Moose density and snow depth both correlated positively to adult moose group size. In contrast, the proportion of females (sex-ratio) was negatively correlated to adult moose group size (Table 3).

**Table 2:** Model selection to predict adult moose group size in Sweden applied to 1335 observations of moose groups from survey data collected in 2006, 2009, and 2011

Variable	-logLik	AIC	N parameters	∆_i_	ω_i_
Moose density + Snow depth + Sex-ratio + *I*	1954.00	3917.99	5	0	0.65
Wolf presence + Moose density + Snow depth + Sex-ratio + *I*	1953.77	3919.55	6	1.60	0.30

Models are shown in order of decreasing rank with model log-likelihood (logLik), number of model parameters (N parameters), Akaike’s information criterion (AIC), AIC differences (∆_i_) and AIC weights (ω_i_)

*I* = spatial autocorrelation

**Table 3:** Coefficients estimates (β) and 85% confidence intervals (CI) of moose density, snow depth, sex-ratio and spatial autocorrelation to predict adult moose group size (*n* = 1335) in Sweden from aerial counts 2006, 2009, and 2011

Variable	B	85% CI Lower - Upper
Moose density	0.017	0.0082 - 0.025
Snow depth	0.0037	0.00011 - 0.0063
Sex-ratio	-1.64	-2.52 - 0.77
Spatial autocorrelation (*I*)	0.44	0.38 - 0.51
Wolf presence (inside/outside territories)	-0.013	-0.13 - 0.049

We then used the same set of 12 models to evaluate the performance of explanatory variables for each moose group category separately. For four of the five different categories (females + calves, mixed group + calves, mixed group no calves, and females), the NULL model (i.e., intercept only) was the most parsimonious to explain variation in moose group size. In contrast, the best model to explain variation in male group size included both moose density and the presence of wolves and these variables together explained 13% of the variation in male group size (Table 4). In these models, wolf presence was positively correlated to male group size. However, two other models including a combination of wolf presence, moose density, snow depth and sex-ratio also received high empirical support (Table 4). Moose density and snow depth were both positively correlated to group size of males whereas sex-ratio had the opposite effect (Table 5). The most important variable to predict male group size was moose density, followed by wolf presence, snow depth and sex-ratio (Table 6).

**Table 4:** Top models to predict male group size in Sweden applied to 225 observations of male moose groups from aerial survey data collected in 2006, 2009, and 2011

Model	-logLik	AIC	N parameters	∆_i_	ω_i_
Wolf presence + Moose density	321.04	648.08	3	0	0.41
Moose density + Snow depth + Sex-ratio	320.46	648.91	4	0.83	0.27
Wolf presence + Moose density + Snow depth + Sex-ratio	319.75	649.51	5	1.40	0.20

Models are shown in order of decreasing rank with model log-likelihood (logLik), number of model parameters (N parameters), Akaike’s information criterion (AIC), AIC differences (∆_i_) and AIC weights (ω_i_)

**Table 5:** Coefficients estimates (β) and 85% confidence intervals (CI) of moose density, snow depth, sex-ratio and wolf presence to predict male group size in Sweden from aerial survey data collected in 2006, 2009, and 2011

		85% CI	
Variable	B	Lower	Upper
Moose density	0.039	0.018	0.060
Snow depth	0.0028	-0.0014	0.012
Sex-ratio	-1.92	-6.41	-0.98
Wolf presence	0.20	0.085	0.50

**Table 6:** Relative importance of moose density, wolf presence, snow depth and sex-ratio to predict male group size in Sweden from aerial survey data collected in 2006, 2009, and 2011

Variable	Importance	*N*
Moose density	0.89	5
Wolf presence	0.70	5
Snow depth	0.52	5
Sex ratio	0.48	4

*N* indicates the number of models in which the variable was used

## Discussion

In this study, moose did not form large groups and mostly stayed solitary or in small groups consisting of two to three animals. The results show, that wolf re-establishment in Sweden has not resulted in an overall change in social grouping behaviour among moose. Nor did we find any support that females with calves, i.e., the category most vulnerable to predation [38, 39], change their grouping behaviour. In fact, other variables such as moose density, snow depth and adult sex ratio of the group seemed to be more important for the overall pattern of grouping behaviour. However, when analyzing moose categories separately, males were found to form larger groups inside wolf territories.

In the light of other studies of ungulates presenting evidence of changed grouping behaviour as a response to predation risk, especially for females with calves [3, 24], our results may at first glance seem unexpected. Young of the year are more susceptible to direct predation [24, 38, 39] and female with calves should thereby be expected to respond more strongly to an increase in predation risk [62–64]. Although increased grouping behaviour in general is viewed as an effective anti-predator response, the plasticity of this behaviour is likely to be both species- and context dependent. For instance, elk (*Cervus elaphus*) formed smaller herds when wolves were present, which is thought to reduce the likelihood of being detected by wolves [24]. Another study suggests that elk may adopt different strategies to minimize predation risk whereby they either choose to live in small herds that are rarely encountered by wolves, or they choose to live in large herds that reduce their predation risk by dilution and many eyes scanning [17]. Solitary or small groups are strategies that have been suggested to be best suited for concentrate selectors and animals in forested terrain and close to cover [18, 65] and specifically for animals less likely to benefit of group living because of the high probability of being attacked by a selective predator once encountered by a predator [18]. However, as the grouping pattern of females with calves was not at all related to the presence of wolves in our study this observation provides support for that the grouping behaviour of female moose in Scandinavia is more a result of other factors affecting the benefit of grouping, e.g., foraging and competition for access to food [65]. Further, as food competition is likely to affect low ranked individuals more [29, 30] it is also possible that these costs may differ among categories of moose, i.e., a higher cost for females with calves than for males, which may explain why females with calves did not exhibit a change in grouping behaviour while males did. Similarly, Creel [24] showed that elk male groups increased in size when wolves were present contrary to mixed herds that decreased in size. Given our data, we cannot address the underlying mechanism for this divergent pattern among moose categories in our study. However, Creel [24] suggested that poor condition of males post rut forced elk males to spend more time foraging and less time being vigilant and thereby they should benefit more than other animals from forming larger groups. Early detection of approaching wolves has been shown to increase survival of moose targeted by wolves [27, 66]. Moreover, a large number of males in the Swedish moose population are young (e.g., yearlings) mainly because of high turnover rate in the population due to intensive harvest strategies (and especially so for males) [42, 67] and therefore these groups may mainly consists of young males similar to “bachelor herds” in other species of ungulates [9]. Yearlings are the second most common age class among moose killed by wolves in Scandinavia but still less vulnerable than calves (Sand et al. unpubl. data).

Our results are partly in line with several recent studies that have investigated changes in moose behaviour as a response to wolf re-colonization in Scandinavia. In general, there is no or only weak support for that moose behaviour has changed with increased wolf predation risk, and if these effects exist they are small relative to the effect of population structure of prey and environmental factors [36, 43, 68–72]. Compared to the other studies investigating behaviorally mediated effects of wolf return in Scandinavia this study may provide some support for a behavioural response though it was not consistent across the entire population.

However, the response by male moose was opposite to the one predicted, i.e., male groups was larger within than outside wolf territories. Similar to Sand et al. [43] and Gervasi et al. [70], we did not find any relationship between the degree of behavioural change and time since establishment of wolf territories. However, that social population structure may be important for the behaviour of ungulates as well as other factors than predation risk such as habitat type, population density, snow conditions, and the distribution and availability of food is well known [22, 73, 74]. For example, a positive relationship between group size and population density is documented [17, 65, 73] as is the relation with snow [74]. Increased snow cover decreases food availability by creating limited and irregular food patches but also restricts movements of moose [74]. Thus, moose individuals tend to group more in areas that provide high energy intake and where the costs of mobility may be reduced by taking advantage of the tracks made by conspecifics [4, 23, 74]. Moreover, habitat type and composition can affect grouping behaviour and for example in open habitats, ungulates tend to form larger groups [18, 31, 32]. However, moose do not change habitat selection in relation to predation risk by wolves in Sweden [69]. Also in the current study, moose used the habitats similarly inside and outside wolf territories (forested areas (inside 95% of observed groups, outside 96%), wetlands and lakes (inside 4%, outside 3%) and other (urban and agriculture; inside 1%, outside 1%)). Furthermore, the habitat composition inside and outside wolf territories were similar (dominated by forested areas (inside 74% of total area, outside 80%) wetlands and lakes (inside 17%, outside 18%) and other (urban and agriculture; inside 9%, outside 2%)).

## Conclusions

The results did not give support for that wolf recolonization in Scandinavia has resulted in an overall change in moose grouping behaviour. Nor could we confirm our two predictions that females with calves should be the category most prone to change their grouping behaviour and that moose grouping behaviour was related to the time since wolf territory establishment. Rather, the results showed a sex specific difference in social grouping in relation to wolf presence where males group more in areas with wolves. However, even for this moose category population and environmentally related variables was also important for the pattern of grouping. If indeed wolf-induced effects on behavior do exist in the moose population, they may be difficult to discern because the effects from population and environmental factors may be much stronger and thus conceal any subtle change in anti-predator behaviour. These results suggest that caution should be taken as to generalize about the effects of returning predators on the grouping behaviour of their prey.

## Additional file

Additional file 1: Table S1. 12 *a priori* models used to explain moose grouping behaviour in Sweden from aerial survey data collected in 2006, 2009, 2011. Models are shown in order of decreasing rank with model log-likelihood (-logLik), Akaike’s information criterion (AIC), AIC differences (∆_i_) and AIC weights (ω_i_) and deviance. (DOCX 14 kb)
