# Supplementary material for: Group or ungroup – moose behavioural response to recolonization of wolves
Source: Front Zool. 2017 Feb 17;14:10. doi: 10.1186/s12983-017-0195-z (PMC5316190; doi:10.1186/s12983-017-0195-z)
Supplement: Additional file 1: Table S1. — 12 a priori models used to explain moose grouping behaviour in Sweden from aerial survey data collected in 2006, 2009, 2011. Models are shown in order of decreasing rank with model log-likelihood (-logLik), Akaike’s information criterion (AIC), AIC differences (∆i) and AIC weights (ωi) and deviance. (DOCX 14 kb) [file 12983_2017_195_MOESM1_ESM.docx]

Supplemental Table 1. 12 *a priori* models used to explain moose grouping behaviour in Sweden from aerial survey data collected in 2006, 2009, 2011. Models are shown in order of decreasing rank with model log-likelihood (-logLik), Akaike’s information criterion (AIC), AIC differences (∆_i_) and AIC weights (ω_i_) and deviance.

Variables -logLik AIC ∆_i_ ω_i_ Deviance

Moose density + Snow depth + Sex ratio + *I* 1954.00 3917.99 0 0.65 785.51

Moose density + Snow depth + Sex ratio + 1953.77 3919.55 1.56 0.3 785.06

Wolf presence + *I*

Moose density + Snow depth 1958.37 3924.74 6.74 0.02 794.25

Wolf presence + Moose density 1958.87 3925.75 7.76 0.01 795.26

Moose density 1960.37 3926.75 8.76 0.01 798.26

Wolf presence + Snow depth 1959.65 3927.3 9.31 0.01 796.82

Snow depth 1961.03 3928.07 10.07 0 799.58

Sex ratio 1962.57 3931.14 13.15 0 802.65

Wolf presence 1963.21 3932.42 14.43 0 803.93

Wolf presence + Sex ratio 1962.35 3932.69 14.7 0 802.21

Spatial autocorrelation 1965.21 3934.42 16.43 0 807.94

Intercept 2010.33 4022.66 104.66 0 898.17

*I* = spatial autocorrelation
